# Chinese adaptation and psychometric evaluation of the competence scale of actions of nurses in emergencies among emergency nurses

**DOI:** 10.3389/fmed.2026.1874004

**Published:** 2026-06-29

**Authors:** Zhang Li, Xiao Qian, Zhang Linyuan, Zhang Meixia, Wang Lulu, Li Junjie, Hu Xuehui

**Affiliations:** 1 Department of Emergency Medicine, Xijing Hospital, The Fourth Military Medical University, Xi’an, Shaanxi, China; 2 Department of Nursing, Xijing Hospital, The Fourth Military Medical University, Xi’an, Shaanxi, China; 3 School of Nursing, Air Force Medical University, Xi’an, Shaanxi, China

**Keywords:** emergency nursing, emergency response capability, nurses, psychometric evaluation, scale

## Abstract

**Objective:**

To translate the Competence Scale of Actions of Nurses in Emergencies (CSANE) into Chinese and evaluate its reliability and validity.

**Methods:**

A total of 465 emergency department nurses from 10 tertiary hospitals in Xi’an, Shaanxi Province, China, participated in this study. The original CSANE was translated following Brislin’s translation model, culturally adapted through expert consultation and pilot testing, and finalized as the Chinese CSANE. Reliability was evaluated using Cronbach’s alpha coefficients and split-half reliability. Validity was assessed through content validity, exploratory factor analysis, confirmatory factor analysis, convergent validity, and discriminant validity.

**Results:**

The Chinese CSANE comprises 7 dimensions and 78 items. Exploratory factor analysis extracted 7 common factors, accounting for 76.919% of cumulative variance. In confirmatory factor analysis, CMIN/DF was 1.736, RMR was 0.021, IFI was 0.953, TLI was 0.952, CFI was 0.953, and RMSEA was 0.040, whereas GFI and AGFI were 0.794 and 0.781, respectively. The AVE values ranged from 0.714 to 0.845, and the CR values ranged from 0.910 to 0.989. However, discriminant validity was insufficient because several inter-factor correlations exceeded the square roots of AVE. The total Cronbach’s alpha coefficient was 0.994, and the split-half reliability was 0.975.

**Conclusion:**

The Chinese CSANE showed good internal consistency and acceptable structural validity among emergency nurses. However, the insufficient discriminant validity and high Cronbach’s alpha coefficient suggest potential overlap among some dimensions and items. Further multicenter studies and item refinement are needed to improve its applicability and measurement precision.

**Contribution to patients or public:**

The Chinese CSANE may help nursing administrators understand emergency nurses’ competency levels and training needs, and provide a basis for targeted training and management.

## 1 Introduction

As the primary force in emergency response (1, 2), emergency nurses require not only solid professional knowledge and proficient technical skills but also critical competencies such as rapid assessment, precise decision-making, and multitasking to address the needs of critically ill patients (3, 4). Their emergency response capabilities directly impact treatment efficiency and patient outcomes, making scientific assessment of these abilities crucial for nursing quality management (3). While internationally validated emergency competency scales exist, China lacks standardized tools adapted to its cultural and healthcare context. Clinical assessments often rely on subjective judgments, leading to significant biases. This study aimed to translate and culturally adapt the CSANE into Chinese and evaluate its psychometric properties among emergency nurses in China, so as to provide a reference for emergency nursing competency assessment and training.

## 2 Background

Emergency response capability constitutes a vital component of hospital emergency management systems and serves as a core indicator for evaluating emergency care standards (5, 6). This capability specifically refers to a nurse’s comprehensive ability to rapidly assess patient conditions and make decisions based on professional expertise and clinical judgment during critical situations, while dynamically monitoring patient status changes and efficiently managing emergencies (7). As a vital dimension of specialized emergency nursing competence, emergency response capability encompasses multiple aspects including crisis recognition, risk anticipation, team collaboration, and psychological adaptation, directly impacting patient treatment outcomes and nursing service quality (8, 9). Previous studies indicate a close correlation between emergency nurses’ response capabilities and patient safety (10), underscoring the critical importance of scientifically and effectively evaluating these competencies.

Currently, research on emergency response capabilities among Chinese emergency nurses primarily consists of reviews and status surveys. Commonly used assessment tools include the Emergency Nurse Public Health Emergency Response Scale developed by Zhang (7) and the Non-Emergency Nurse Public Health Emergency Response Scale developed by Yang (11). Both scales comprise 7 dimensions and 37 items, yet they exhibit limitations: the former lacks theoretical grounding, being developed solely through literature review, with its reliability and validity requiring further validation; the latter primarily targets non-emergency nurses, failing to meet the specific assessment needs for emergency nurses’ competence.

The Competence Scale of Actions of Nurses in Emergencies (CSANE) was initially developed by Brazilian scholar Holanda et al. (12), validated in Holanda et al. (13), and widely adopted among emergency nurses globally after translations in South Korea and other countries by 2024. Its reliability and validity have been previously examined (14). After obtaining authorization from the original author, this study adapted the CSANE into Chinese and evaluated its psychometric properties among Chinese emergency nurses.

## 3 Methods

### 3.1 Design

This was a cross-sectional methodological study involving translation, cultural adaptation, and psychometric evaluation of the CSANE.

### 3.2 Participants

From June to July 2025, 465 emergency department nurses from 10 tertiary hospitals in Xi’an, Shaanxi Province, China, were selected using convenience sampling. Inclusion criteria: (1) Emergency department nurses with ≥1 year of work experience; (2) Registered emergency nurses with secondary technical school education or above; (3) Voluntary participation in this study. Exclusion criteria: (1) Interns or trainees; (2) Nurses absent due to leave, resignation, etc. According to factor analysis requirements (15), the sample size should be at least 5–10 times the number of scale items. The CSANE contains 78 items; therefore, the required sample size was 390–780. Considering a 10% non-response rate, the target sample size was approximately 429–858. A total of 475 questionnaires were distributed, and 465 valid questionnaires were returned, meeting the minimum requirement for factor analysis.

### 3.3 Scale introduction

The CSANE scale comprises 7 dimensions and 78 items: Professional Practice (33 items), Work Relationships (19 items), Positive Challenge (10 items), Guiding Actions (7 items), Constructive Behavior (2 items), Professional Advancement (4 items), and Adapting to Change (3 items). A 5-point Likert scale is used, with scores ranging from 1 (disagree) to 5 (strongly agree). All items are scored in the positive direction. The total score ranges from 78 to 390, with higher scores indicating stronger emergency response capabilities among emergency nurses. The original scale showed good psychometric properties, with a Cronbachs’*α* coefficient of 0.980.

### 3.4 Scale translation and content validity assessment

Prior to testing the psychometric properties of the Chinese CSANE, translation and content validity assessments were completed. After obtaining permission and research guidance from the original author of the CSANE, we translated the original version into Chinese, followed by translation, back-translation, cultural adaptation, and pilot studies. (1) Translation: The Brislin translation model (16) was applied to localize the scale. Direct translation: Two Master of Nursing graduates independently translated the original scale into Chinese, producing versions C1 and C2. Both translators had experience in reading English nursing literature and were familiar with emergency nursing terminology. A nursing doctoral scholar proficient in English and relevant theoretical knowledge compared and synthesized the two translations, and the Chinese version C3 was formed after discussion and consensus. (2) Back-Translation: Two additional nursing master’s degree holders, who were unfamiliar with the original scale, independently back-translated the Chinese version into English versions E1 and E2. A nursing doctoral scholar compared the back-translated versions with the original scale, discussed items with inconsistent wording with the research team, and produced the back-translated version E3. (3) Author Review: Both English version E3 and Chinese version C3 were submitted to the original author for verification. Discrepancies in wording were addressed through consultation and negotiation to reach consensus, ultimately forming the Chinese version C4. (4) Cross-cultural adaptation: Conducted using an expert letter inquiry method. Expert inclusion criteria: Master’s degree or higher; Associate Senior title or above; 10 + years of experience in emergency medicine or nursing management-related fields. Nine experts were invited, including seven nursing specialists and two emergency medicine professors. Among them, three held doctoral degrees and six held master’s degrees. Their ages ranged from 33 to 60 years (mean 47.08 ± 7.77), with work experience spanning 10 to 37 years (mean 21.17 ± 9.20). Through cultural adaptation, the Chinese version C5 was finalized. (5) Pilot study: Using convenience sampling, 30 nurses from the emergency departments of 10 tertiary hospitals in Xi’an, Shaanxi Province, participated in a pre-survey. Based on feedback and results from the pre-survey participants, items that were difficult to understand were further revised to form the Chinese version C6 for the formal survey.

### 3.5 Data collection and quality control methods

Data were collected using a nurse demographic questionnaire and the Chinese version of the CSANE scale. The survey was administered via Wenjuanxing, with questionnaires distributed online through each hospital’s nursing department. Each IP address or mobile device could complete and submit the questionnaire only once to prevent duplicate or proxy responses. All items were set as single-choice questions; multiple selections or omissions were invalid. Complete submission required all items to be fully answered to prevent missing responses. All questionnaires were completed and submitted online. After data collection, two researchers screened the responses for completeness and validity. Questionnaires completed in <2 min were excluded because this duration was considered insufficient to read and respond to all 78 items carefully based on the pilot survey. Obvious response patterns were defined as selecting the same option for all items, repeated straight-line responses, or highly regular alternating responses. A total of 475 questionnaires were distributed, and 465 valid responses were collected, yielding a valid response rate of 97.89%.

### 3.6 Statistical methods

Data entry was double-checked by two individuals using Excel software, and data analysis was performed using SPSS 25.0 and AMOS 24.0 software. (1) The critical ratio method was used to assess item discrimination, evaluating the ability of scale items to differentiate between groups. The questionnaire responses from 465 participants were ranked by total score from highest to lowest. The top 27% were assigned to the high-score group, and the bottom 27% were assigned to the low-score group. An independent samples t-test was conducted on the two groups. If *p* < 0.05, it indicated good discrimination between scale items. (2) Correlation analysis was used to assess the relationship between individual item scores and the total scale score. Items with a correlation coefficient *r* < 0.4 or *p* ≥ 0.05 were removed. (3) Cronbach’s alpha coefficient and split-half reliability were used to evaluate the internal consistency of the Chinese CSANE. A Cronbach’s alpha or split-half reliability coefficient >0.700 indicates good reliability, 0.600–0.700 indicates moderate reliability, and <0.600 indicates that the scale requires modification. (4) The validity of the Chinese CSANE was evaluated using content validity and structural validity. Content validity: Nine experts evaluated the scale’s content validity, including item-content validity index (I-CVI) and scale-content validity index (S-CVI). The scale-content validity index was expressed as the average S-CVI (S-CVI/Ave). Content validity was assessed using a 4-point Likert scale: no correlation = 1 point, weak correlation = 2 points, moderate correlation = 3 points, strong correlation = 4 points. The content validity of each item (I-CVI) was calculated as the number of experts scoring 3 or 4 divided by the total number of experts evaluating that item. The scale-level content validity (S-CVI/Ave) was then derived. An I-CVI value >0.78 and S-CVI/Ave ≥ 0.90 were set as the acceptance criteria. Structural validity: Factor analysis was used to evaluate the structural validity of the Chinese CSANE while retaining the original seven-factor structure. The scale underwent KMO and Bartlett’s sphericity tests. When KMO > 0.8 and Bartlett’s *χ*^2^ value reached statistical significance (*p* < 0.05), factor analysis was deemed appropriate. Principal component analysis and maximum variance orthogonal rotation were employed, with item loadings >0.450 serving as criteria for item retention and factor assignment. In confirmatory factor analysis, multiple fit indices are typically selected, including the chi-square/degrees-of-freedom ratio (*χ*^2^/df), Normative Fit Index (NFI), Incremental Fit Index (IFI), Tucker–Lewis Index (TLI), Comparative Fit Index (CFI), Goodness-of-Fit Index (GFI), and Root Mean Square Error of Approximation (RMSEA) to evaluate model fit. Acceptance criteria are set as follows: *χ*^2^/df < 3, NFI > 0.80, IFI > 0.90, TLI > 0.90, CFI > 0.90, GFI > 0.90, and RMSEA < 0.08.

### 3.7 Ethical considerations

This study was approved by the Ethics Committee of the First Affiliated Hospital of Air Force Medical University (approval no.: KY20232334-C-1). All participants provided informed consent and voluntarily participated. Data collection occurred within the hospital setting, with strict protection of participants’ privacy and anonymity. All participants were informed that data would be kept strictly confidential.

## 4 Results

### 4.1 Demographic characteristics

Among 465 emergency nurses, 56 were male and 409 were female. By age: 175 were <30 years old, 191 were 30–40 years old, 74 were 41–50 years old, and 25 were >50 years old. Educational background: 23 had secondary technical school education, 114 held associate degrees, 300 held bachelor’s degrees, and 28 held master’s degrees or higher. Monthly income: 152 earned <5,000 yuan, 212 earned 5,000–10,000 yuan, and 101 earned >10,000 yuan. Professional titles: 135 were nurses, 178 were senior nurses, 124 were supervisor nurses, and 28 were deputy chief nurses or above. Work experience: 182 had <5 years, 149 had 5–10 years, 74 had >10–<15 years, and 60 had ≥15 years.

### 4.2 Scale content revision

Based on findings from translation, back-translation, cultural adaptation, pilot studies, and research team discussions, corresponding modifications were made to the scale content. Specific revisions are as follows: (1) Item 2: “Are limited medical resources ambiguously defined?” was revised to “Can you reasonably utilize limited medical resources (staff/equipment/instruments/medications/facilities) during work?”; (2) Item 55: “Medical principles” was too broad and was revised to “Do you consistently adhere to the principles of prioritizing life, saving lives, and medical equality?” (3) Item 28: “When guiding nurses in providing patient care, do you carefully consider and assume risks?” was incomplete and revised to “When implementing patient care, do you carefully consider and comprehensively assess risks, anticipating necessary risks?” (4) Item 39: The expression “existing media” did not align with Chinese usage and was revised to “existing communication channels.” (5) Items 65, 68, 69: “Can you be mindful of stimuli from people/equipment/environment?”—The term “stimuli” does not conform to Chinese usage. The expression was revised to “Can you keenly perceive safety hazards in the environment/from patients/in equipment?” (6) Item 1 was revised from “Do you have confidence in resolving sudden emergencies?” to “Do you have confidence in resolving sudden emergencies encountered at work? Examples include mass casualty incidents, burn injuries, or unidentified pre-hospital patients,” to clarify the clinical context.

### 4.3 Psychometric properties

#### 4.3.1 Item analysis results

The total scores of the 78 items in the Chinese CSANE were divided into high-score and low-score groups. An independent samples t-test revealed significant differences between the two groups on all items (*p* < 0.001), indicating good discriminative power among the scale items. Correlation analysis revealed that the correlation coefficients between each item score and the total scale score ranged from 0.730 to 0.892, with all coefficients >0.4 and *p* < 0.001. This indicates that each item effectively reflects the themes measured by the scale. Based on the item analysis results, all items of the Chinese CSANE were retained.

#### 4.3.2 Content validity results

**Figure 1:**
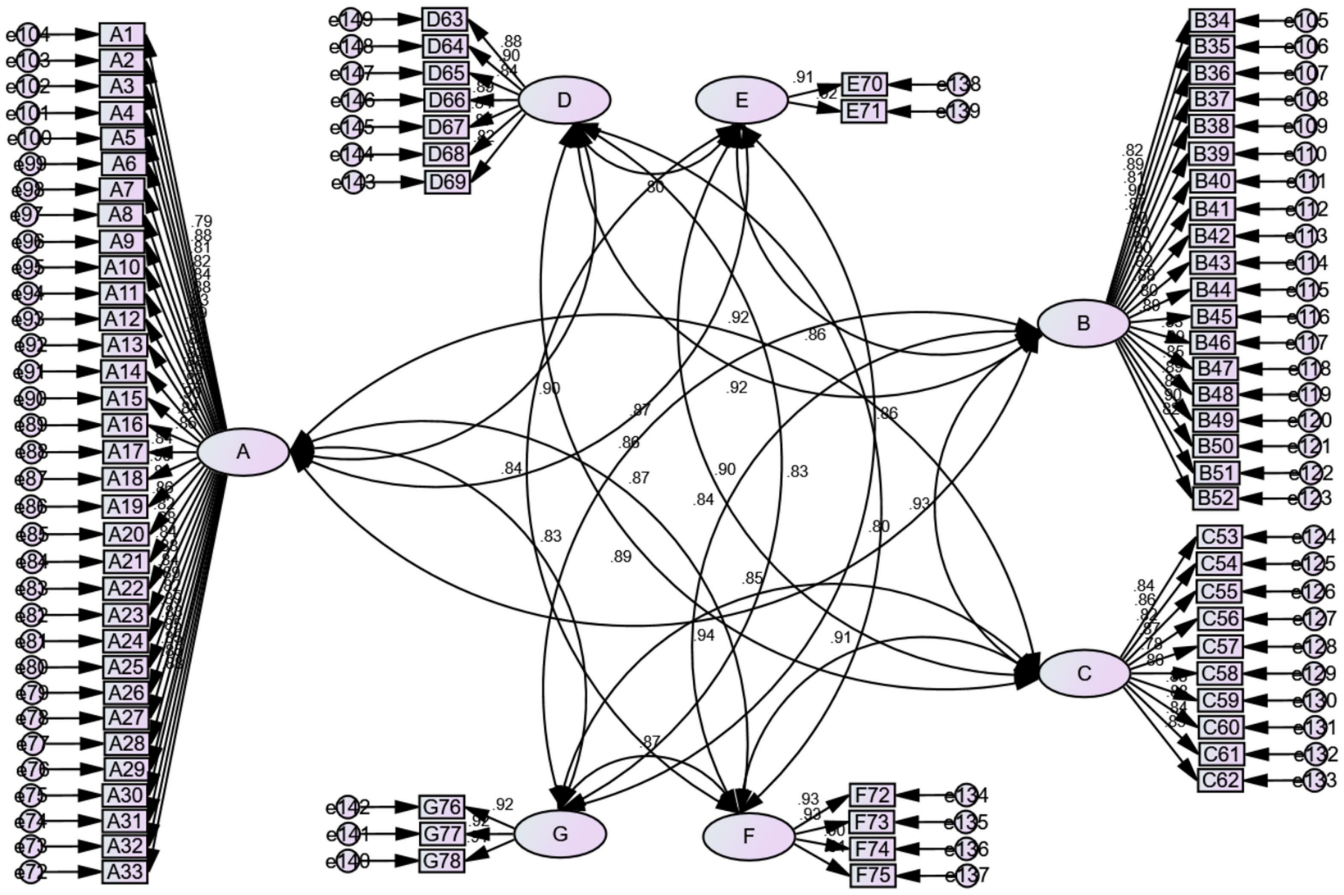
Confirmatory factor analysis model of the Chinese CSANE.

Based on expert evaluations, the I-CVI values for the Chinese CSANE ranged from 0.77 to 1.00, and the S-CVI/Ave was 0.93. Although some items had relatively low I-CVI values, they were retained after expert discussion because they were considered relevant to the construct of emergency nursing competence (Figure 1).

#### 4.3.3 Exploratory factor analysis

Factor analysis was conducted on the Chinese CSANE among 465 emergency nurses. The KMO value was 0.989, and Bartlett’s sphericity test yielded *χ*^2^ = 45669.989 (*p* < 0.001), indicating that the data were suitable for factor analysis. For this Chinese adaptation, principal component analysis (PCA) with maximum variance orthogonal rotation was employed, fixing the number of factors at seven. The cumulative variance explained reached 76.919%. All item loadings on corresponding common factors were ≥0.650, ranging from a minimum of 0.675 to a maximum of 0.875. The seven-factor solution generally corresponded to the original scale structure, providing preliminary support for the structural validity of the Chinese CSANE. The rotated factor matrix is presented in Table 1.

**Table 1:** Exploratory factor analysis results of the Chinese CSANE (*n* = 465).

Item	Common factor						
	Factor 1 Professional practice	Factor 2 Work relationships	Factor 3 Positive challenge	Factor 4 Guiding actions	Factor 5 Adapting to change	Factor 6 Professional advancement	Factor 7 Constructive behavior
A25	**0.666**	0.318	0.301	0.251	0.074	0.211	0.056
A26	**0.663**	0.411	0.288	0.219	0.145	0.132	0.164
A6	**0.658**	0.376	0.234	0.26	0.207	0.154	0.135
A22	**0.657**	0.326	0.308	0.294	0.221	0.067	0.154
A18	**0.656**	0.418	0.266	0.246	0.223	0.091	0.136
A10	**0.656**	0.347	0.224	0.219	0.228	0.09	0.222
A8	**0.654**	0.348	0.212	0.324	0.231	0.174	0.164
A28	**0.653**	0.374	0.312	0.242	0.236	0.082	0.138
A15	**0.652**	0.292	0.259	0.192	0.236	0.216	0.124
A14	**0.651**	0.347	0.286	0.242	0.277	0.21	0.134
A23	**0.649**	0.399	0.25	0.142	0.211	0.201	0.03
A5	**0.648**	0.301	0.338	0.22	0.082	0.169	0.179
A7	**0.648**	0.33	0.19	0.252	0.206	0.271	−0.007
A9	**0.645**	0.303	0.185	0.331	0.082	0.274	−0.008
A2	**0.644**	0.302	0.273	0.281	0.219	0.239	0.134
A4	**0.644**	0.222	0.304	0.244	0.184	0.184	0.155
A12	**0.643**	0.346	0.297	0.29	0.189	0.17	0.096
A33	**0.64**	0.367	0.273	0.282	0.183	0.184	0.129
A24	**0.639**	0.392	0.281	0.266	0.179	0.101	0.184
A29	**0.639**	0.326	0.286	0.284	0.152	0.108	0.035
A16	**0.635**	0.367	0.261	0.224	0.226	0.081	0.217
A21	**0.633**	0.234	0.311	0.275	0.109	0.202	0.157
A32	**0.628**	0.392	0.214	0.249	0.27	0.044	0.126
A31	**0.621**	0.383	0.244	0.278	0.267	0.072	0.032
A27	**0.619**	0.255	0.33	0.256	0.252	0.111	0.063
A20	**0.614**	0.386	0.257	0.296	0.254	0.013	0.141
A11	**0.614**	0.285	0.304	0.243	0.097	0.159	0.252
A3	**0.613**	0.347	0.244	0.213	0.09	0.281	0.086
A17	**0.609**	0.306	0.244	0.267	0.233	0.136	0.248
A13	**0.608**	0.33	0.276	0.214	0.191	0.08	0.212
A30	**0.605**	0.515	0.173	0.211	0.243	−0.016	0.145
A1	**0.605**	0.141	0.257	0.446	0.046	0.113	0.331
A19	**0.603**	0.342	0.31	0.188	0.278	0.091	−0.034
B49	0.417	**0.619**	0.282	0.233	0.273	0.19	0.128
B43	0.427	**0.606**	0.281	0.28	0.219	0.143	0.112
B41	0.437	**0.595**	0.322	0.286	0.198	0.171	0.138
B37	0.448	**0.593**	0.239	0.261	0.252	0.211	0.202
B51	0.434	**0.585**	0.268	0.29	0.274	0.125	0.199
B39	0.46	**0.579**	0.303	0.291	0.222	0.114	0.161
B44	0.373	**0.574**	0.256	0.341	0.119	0.072	0.097
B34	0.407	**0.571**	0.234	0.25	0.221	0.132	0.125
B46	0.411	**0.569**	0.33	0.196	0.149	0.211	0.087
B45	0.431	**0.569**	0.313	0.289	0.196	0.205	0.152
B42	0.395	**0.567**	0.306	0.281	0.064	0.229	0.105
B36	0.375	**0.562**	0.241	0.253	0.243	0.152	0.122
B48	0.418	**0.557**	0.275	0.286	0.181	0.193	0.109
B52	0.416	**0.554**	0.242	0.293	0.272	0.101	0.015
B47	0.447	**0.553**	0.321	0.292	0.24	0.178	0.128
B40	0.341	**0.551**	0.322	0.278	0.1	0.229	0.159
B35	0.457	**0.547**	0.298	0.329	0.241	0.16	0.1
B50	0.394	**0.534**	0.249	0.298	0.217	0.161	0.178
B38	0.431	**0.521**	0.342	0.273	0.207	0.176	0.172
C57	0.322	0.214	**0.697**	0.251	0.133	0.14	0.102
C59	0.352	0.313	**0.622**	0.208	0.185	0.149	0.221
C60	0.38	0.32	**0.606**	0.29	0.261	0.166	0.078
C58	0.392	0.328	**0.578**	0.286	0.218	0.167	0.12
C55	0.392	0.467	**0.567**	0.169	0.158	0.04	0.073
C56	0.414	0.414	**0.545**	0.261	0.247	0.039	0.156
C53	0.352	0.366	**0.544**	0.264	0.217	0.195	0.096
C61	0.39	0.332	**0.52**	0.276	0.148	0.296	0.133
C54	0.415	0.461	**0.506**	0.202	0.202	0.194	0.088
C62	0.442	0.302	**0.481**	0.337	0.31	0.098	0.117
D69	0.351	0.327	0.248	**0.641**	0.152	0.114	0.089
D63	0.395	0.384	0.249	**0.638**	0.194	0.115	0.008
D67	0.387	0.278	0.321	**0.627**	0.199	0.079	0.062
D66	0.392	0.372	0.255	**0.598**	0.203	0.222	0.124
D65	0.351	0.348	0.241	**0.581**	0.183	0.119	0.251
D64	0.393	0.38	0.288	**0.57**	0.259	0.186	0.094
D68	0.404	0.366	0.259	**0.551**	0.207	0.185	0.176
G76	0.335	0.321	0.275	0.272	**0.666**	0.221	0.132
G78	0.332	0.331	0.317	0.232	**0.657**	0.168	0.148
G77	0.378	0.38	0.265	0.269	**0.617**	0.169	0.13
F72	0.377	0.369	0.36	0.243	0.334	**0.52**	0.149
F74	0.357	0.396	0.321	0.235	0.328	**0.509**	0.153
F73	0.388	0.365	0.364	0.245	0.318	**0.501**	0.166
F75	0.334	0.384	0.388	0.295	0.303	**0.461**	0.211
E71	0.432	0.37	0.284	0.201	0.23	0.196	**0.559**
E70	0.43	0.33	0.296	0.206	0.259	0.169	**0.559**

Bold values indicate the highest factor loading for each item, representing the primary dimension to which the item belongs.

#### 4.3.4 Confirmatory factor analysis

Confirmatory factor analysis was conducted using AMOS 26.0 to further examine the seven-factor structure of the Chinese CSANE. The model fit indices are shown in Table 2. The CMIN/DF was 1.736, and the RMR was 0.021. In addition, IFI, TLI, and CFI were 0.953, 0.952, and 0.953, respectively, and RMSEA was 0.040. These indices indicated an acceptable model fit. However, GFI and AGFI were 0.794 and 0.781, respectively, which were below the recommended thresholds. Therefore, although most indices supported the model fit, the CFA results should be interpreted with caution.

**Table 2:** Confirmatory factor model fit indices.

Fit indices	Recommended range		Value	Result
	Excellent	Good		
CMIN/DF	<3	<5	1.736	Excellent
RMR	<0.08	<0.08	0.021	Excellent
GFI	>0.9	>0.8	0.794	Poor
AGFI	>0.9	>0.8	0.781	Poor
NFI	>0.9	>0.8	0.897	Good
RFI	>0.9	>0.8	0.893	Good
IFI	>0.9	>0.8	0.953	Excellent
TLI	>0.9	>0.8	0.952	Excellent
CFI	>0.9	>0.8	0.953	Excellent
RMSEA	<0.08	<0.08	0.04	Excellent

As shown in Table 2, most fit indices met the recommended criteria, including CMIN/DF, RMR, IFI, TLI, CFI, and RMSEA. However, GFI and AGFI were below the recommended thresholds. Therefore, the model fit was considered acceptable but not fully ideal.

As shown in Table 3, the standardized factor loadings for all items were above 0.50. The AVE values for the seven dimensions were 0.725, 0.735, 0.714, 0.746, 0.835, 0.839, and 0.845, respectively. The CR values were 0.989, 0.981, 0.961, 0.954, 0.910, 0.954, and 0.942, all of which exceeded 0.70. These results supported acceptable convergent validity and composite reliability.

**Table 3:** Results of confirmatory factor analysis.

Path			Standardized coefficient	AVE	CR
A1	←	A	0.793	0.725	0.989
A2	←	A	0.876		
A3	←	A	0.809		
A4	←	A	0.823		
A5	←	A	0.842		
A6	←	A	0.88		
A7	←	A	0.83		
A8	←	A	0.892		
A9	←	A	0.81		
A10	←	A	0.858		
A11	←	A	0.817		
A12	←	A	0.88		
A13	←	A	0.821		
A14	←	A	0.902		
A15	←	A	0.842		
A16	←	A	0.862		
A17	←	A	0.841		
A18	←	A	0.898		
A19	←	A	0.805		
A20	←	A	0.861		
A21	←	A	0.818		
A22	←	A	0.885		
A23	←	A	0.844		
A24	←	A	0.885		
A25	←	A	0.845		
A26	←	A	0.891		
A27	←	A	0.82		
A28	←	A	0.891		
A29	←	A	0.831		
A30	←	A	0.847		
A31	←	A	0.85		
A32	←	A	0.85		
A33	←	A	0.882		
B34	←	B	0.821	0.735	0.981
B35	←	B	0.893		
B36	←	B	0.81		
B37	←	B	0.898		
B38	←	B	0.867		
B39	←	B	0.898		
B40	←	B	0.804		
B41	←	B	0.901		
B42	←	B	0.82		
B43	←	B	0.876		
B44	←	B	0.798		
B45	←	B	0.889		
B46	←	B	0.826		
B47	←	B	0.895		
B48	←	B	0.845		
B49	←	B	0.893		
B50	←	B	0.827		
B51	←	B	0.896		
B52	←	B	0.819		
C53	←	C	0.836	0.714	0.961
C54	←	C	0.865		
C55	←	C	0.823		
C56	←	C	0.875		
C57	←	C	0.784		
C58	←	C	0.862		
C59	←	C	0.835		
C60	←	C	0.876		
C61	←	C	0.839		
C62	←	C	0.849		
D63	←	D	0.879	0.746	0.954
D64	←	D	0.901		
D65	←	D	0.836		
D66	←	D	0.895		
D67	←	D	0.84		
D68	←	D	0.87		
D69	←	D	0.823		
E70	←	E	0.907	0.835	0.910
E71	←	E	0.921		
F72	←	F	0.925	0.839	0.954
F73	←	F	0.926		
F74	←	F	0.899		
F75	←	F	0.914		
G76	←	G	0.923	0.845	0.942
G77	←	G	0.924		
G78	←	G	0.91		

As shown in Table 4, the square roots of AVE for the seven dimensions were 0.85, 0.86, 0.84, 0.86, 0.91, 0.92, and 0.92. Several inter-factor correlations were relatively high, including those between dimensions A and B, B and C, and C and F, with correlation coefficients of 0.944, 0.935, and 0.905, respectively. These correlations exceeded the corresponding square roots of AVE, indicating insufficient discriminant validity and suggesting that some dimensions of the Chinese CSANE may overlap conceptually.

**Table 4:** Discriminant validity of the Chinese CSANE.

Dimensions	A	B	C	D	E	F	G
A	0.725						
B	0.944	0.735					
C	0.915	0.935	0.714				
D	0.9	0.921	0.895	0.746			
E	0.861	0.861	0.845	0.799	0.835		
F	0.871	0.904	0.905	0.862	0.837	0.839	
G	0.834	0.865	0.852	0.826	0.797	0.867	0.845
AVE square root	0.85	0.86	0.84	0.86	0.91	0.92	0.92

A = Professional practice; B = Work relationships; C = Positive challenge; D = Guiding actions; E = Constructive behavior; F = Professional advancement; G = Adapting to change.

#### 4.3.5 Reliability

The Chinese CSANE demonstrated a total Cronbachs’*α* coefficient of 0.994 and a split-half reliability of 0.975. The Cronbach’s α coefficients for each dimension ranged from 0.910 to 0.988, with split-half reliability coefficients ranging from 0.835 to 0.983, all exceeding 0.700.

## 5 Discussion

This study followed Brislin’s (16) translation model to translate and culturally adapt the CSANE into Chinese through translation, back-translation, expert consultation, and pilot testing. The results showed that the Chinese CSANE had good content validity, high internal consistency, and acceptable convergent validity among emergency nurses. The CFA results generally supported the seven-factor structure, although the discriminant validity results suggested that some dimensions were not clearly separated. During the cross-cultural adaptation process, nine experts from emergency nursing, nursing management, and emergency medicine reviewed the scale and provided suggestions to improve its semantic clarity and cultural applicability. For example, the expression related to “stimuli” was revised into terms more commonly used in Chinese emergency nursing practice, such as safety hazards in the environment, patients, or equipment. These revisions helped preserve the meaning of the original scale while making the items easier for Chinese nurses to understand.

Reliability is an important indicator for evaluating the consistency of a scale (17). Cronbach’s alpha coefficient and split-half reliability are commonly used to assess internal consistency in Likert-type scales, and a Cronbach’s alpha coefficient ≥0.9 is generally considered to indicate excellent internal consistency (18). In this study, the total Cronbach’s alpha coefficient of the Chinese CSANE was 0.994, and the split-half reliability was 0.975, indicating a high level of internal consistency. This may be related to the standardized translation and cultural adaptation process, as well as the semantic clarification of several items during expert consultation and pilot testing. However, the very high Cronbach’s alpha coefficient may also reflect similarity among some items. When a scale contains a large number of items and some items measure closely related aspects of the same construct, Cronbach’s alpha may become very high. Combined with the high item-total correlations observed in this study, this finding suggests that some items may overlap in content. Therefore, future research may further examine whether some items can be simplified or merged while retaining the core structure and measurement performance of the scale.

Validity assessment employed exploratory factor analysis. Results showed a KMO value of 0.989 and Bartlett’s sphericity test *χ*^2^ = 45669.989 (*p* < 0.001), supporting the applicability of factor analysis. The extracted seven common factors aligned with the original scale structure, indicating the theoretical framework’s suitability for China’s emergency nursing context. The EFA results showed that the seven-factor structure generally corresponded to the original scale. Some items showed relatively high secondary loadings, which may be related to the close connection among emergency nursing competencies in clinical practice. For example, promptly correcting mistakes at work may involve professional practice, positive challenge, and adaptation to change at the same time. Emergency nursing work often requires nurses to complete assessment, communication, decision-making, and adjustment within a short period of time, so some items may naturally overlap across dimensions. In the CFA, most fit indices reached acceptable levels, suggesting that the seven-factor model had a certain degree of structural support. However, GFI and AGFI were below the recommended thresholds, and several dimensions showed high inter-factor correlations. These findings indicate that the overall structure of the Chinese CSANE is broadly consistent with the original scale, but the boundaries between some dimensions still need further verification in independent samples.

Emergency nurses’ emergency response capability is an important component of emergency care quality (1, 19). Accurate assessment is a prerequisite for developing targeted training programs. At present, most assessment tools for this capability in China are self-developed questionnaires, and some still have limitations in item systematization and psychometric validation. The Chinese CSANE may provide a practical reference for evaluating emergency nurses’ emergency response capability. During cultural adaptation, several lengthy or less familiar expressions in the original scale were revised to improve readability. In the pilot survey, nurses completed the questionnaire in less than 15 min on average, which was shorter than the 30 min reported for the original scale (12). This may reduce the response burden and make the scale more suitable for the busy and time-fragmented environment of emergency departments (20). Nursing managers can use the scale to identify potential weaknesses in emergency decision-making, teamwork, risk awareness, and adaptation to complex clinical situations, thereby supporting more targeted competency training and management strategies in emergency nursing. Future studies may further refine the items and verify whether the dimensional structure of the Chinese CSANE is stable in broader clinical samples.

## 6 Limitations

Several limitations should be considered. Although nurses were recruited from 10 tertiary hospitals in Xi’an, convenience sampling was used, and the sample may not fully represent emergency nurses from different regions, hospital levels, or clinical settings. In addition, the same dataset was used for both exploratory factor analysis and confirmatory factor analysis, which may affect the independence of model validation. This study also did not assess test–retest reliability, so the temporal stability of the Chinese CSANE requires further verification. Moreover, the insufficient discriminant validity and the high Cronbach’s alpha coefficient suggest that some dimensions and items may overlap to a certain extent. Future studies could further verify the scale in larger and more diverse samples, assess its stability over time, and consider item reduction or short-form development while maintaining the core structure of the scale.

## 7 Conclusion

The Chinese version of the CSANE comprises 78 items across seven dimensions. It demonstrated good internal consistency, acceptable content validity, and acceptable convergent validity among emergency nurses. However, the insufficient discriminant validity and the high Cronbach’s alpha coefficient suggest that some dimensions and items may overlap. The scale may provide a reference for assessing emergency nurses’ emergency response capability and identifying training needs. Future studies should further verify its applicability in multicenter samples and refine the items to improve measurement precision.

## Data Availability

The datasets presented in this study can be found in online repositories. The names of the repository/repositories and accession number(s) can be found in the article/supplementary material.
